# Awake Fiberoptic Intubation for Airway Management in a Patient With Multinodular Goiter and Severe Tracheal Stenosis

**DOI:** 10.7759/cureus.81174

**Published:** 2025-03-25

**Authors:** Rufino Aguilar Sierra, Hunor Székessy

**Affiliations:** 1 Anesthesia, Stadtspital Zürich Triemli, Zürich, CHE

**Keywords:** anesthesia education, difficult airway management, multinodular goiter, recent trends, vv ecmo

## Abstract

Managing difficult airways in patients with complex comorbidities and language barriers requires a strategic, multidisciplinary approach. We present the case of a 60-year-old non-smoking female with a history of multinodular euthyroid goiter causing significant tracheal stenosis, scheduled for an elective procedure. Her sole documented comorbidity was a newly diagnosed diabetes mellitus. No additional health conditions were reported. A notable challenge was the language barrier, as the patient did not speak the local language, necessitating the presence of a translator to facilitate all communication and informed consent. Given the significant tracheal compression observed in imaging studies, an awake fiberoptic intubation was planned to reduce airway compromise risks. This approach allowed for continuous patient cooperation and monitoring, minimizing the chance of sudden airway obstruction. Throughout the procedure, maintaining clear communication and patient cooperation was essential for the safe execution of the intubation. This case emphasizes the importance of thorough preoperative planning and individualized airway management strategies, particularly in patients with significant anatomical challenges. It highlights the need to adapt standard airway techniques to address complex physiological conditions and underscores the value of a methodical approach to ensure safe and successful outcomes in difficult airway cases.

## Introduction

Airway management in patients with multinodular goiter (MNG) and severe tracheal stenosis presents a critical clinical challenge due to the interplay of anatomical distortion and physiological instability [1]. While videolaryngoscopy has become a cornerstone of modern airway management, its utility diminishes in cases of extrinsic tracheal compression, where direct visualization is obscured by thyroid mass displacement [2]. In such scenarios, awake fiberoptic intubation (AFOI) remains indispensable, offering real-time airway assessment while preserving spontaneous ventilation and protective reflexes [1].

The term "high-risk airway" encompasses both anatomical complexity (e.g., tracheal stenosis <5 mm) and physiological derangements such as hypoxemia or hemodynamic compromise [3]. These factors synergistically elevate the risk of peri-intubation cardiovascular collapse, necessitating meticulous planning tailored to the patient’s unique pathophysiology [4].

The integration of advanced rescue strategies, such as extracorporeal membrane oxygenation (ECMO), may further mitigate risks in anatomically and physiologically high-risk airways. However, ECMO remains inaccessible in many centers due to resource constraints, underscoring the need for adaptable protocols that prioritize airway-specific techniques such as awake fiberoptic intubation. This case exemplifies how meticulous planning, even in settings lacking ECMO capabilities, can achieve safe outcomes through multidisciplinary coordination and patient-specific sedation regimens.

## Case presentation

A 60-year-old, non-smoking, Tamil-speaking female presented to the emergency department with hemoptysis and progressive dyspnea (mMRC grade 3) over one year, exacerbated by exertion. Chronic cough with clear sputum was noted, but she denied fever, weight loss, or systemic symptoms. Her medical history included newly diagnosed diabetes mellitus (HbA1c at 7.7%), managed postoperatively with metformin. A significant language barrier necessitated translation by her daughter for all interactions.

**Figure 1:**
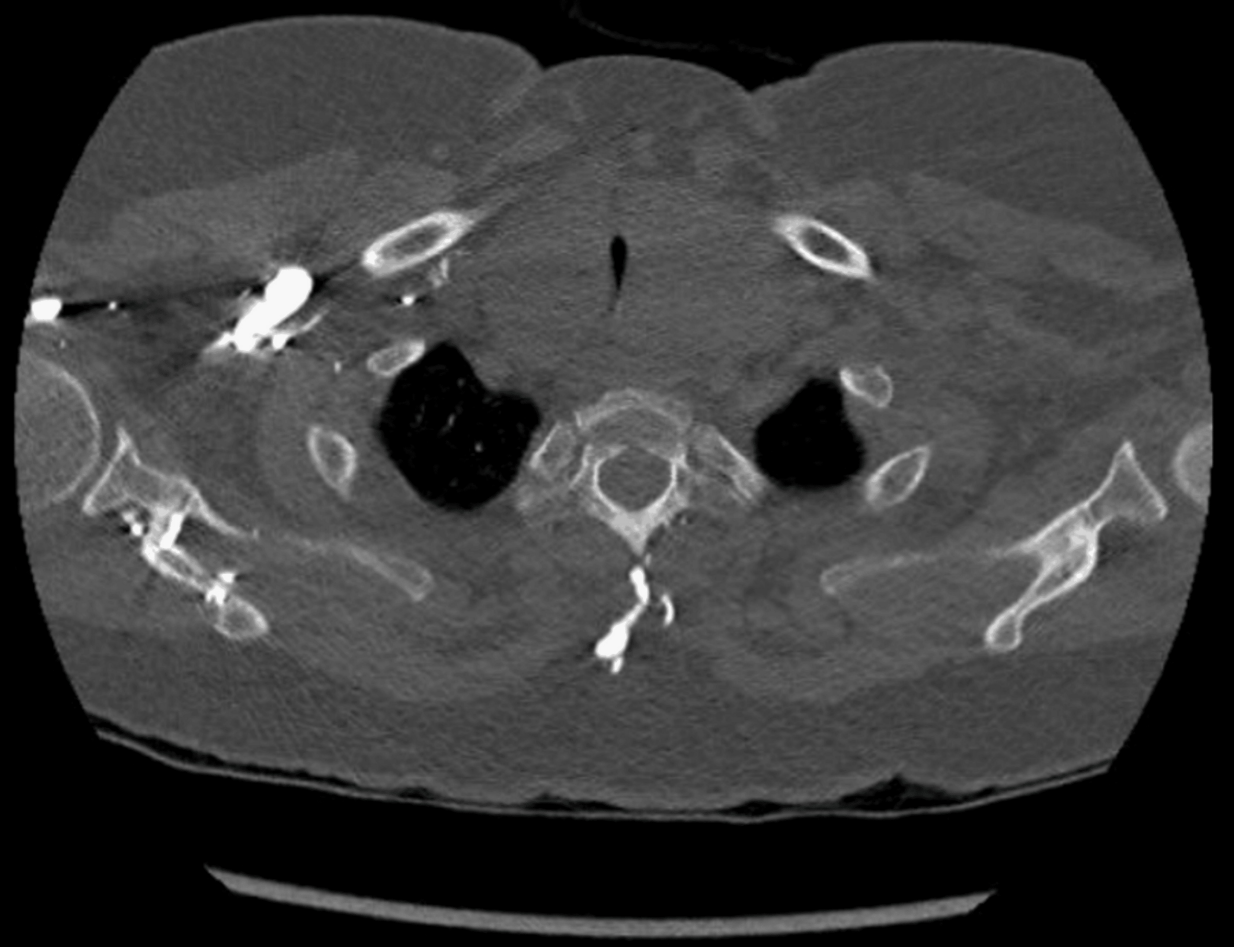
CT image showing a pronounced goiter on both sides with significant tracheal stenosis

**Figure 2:**
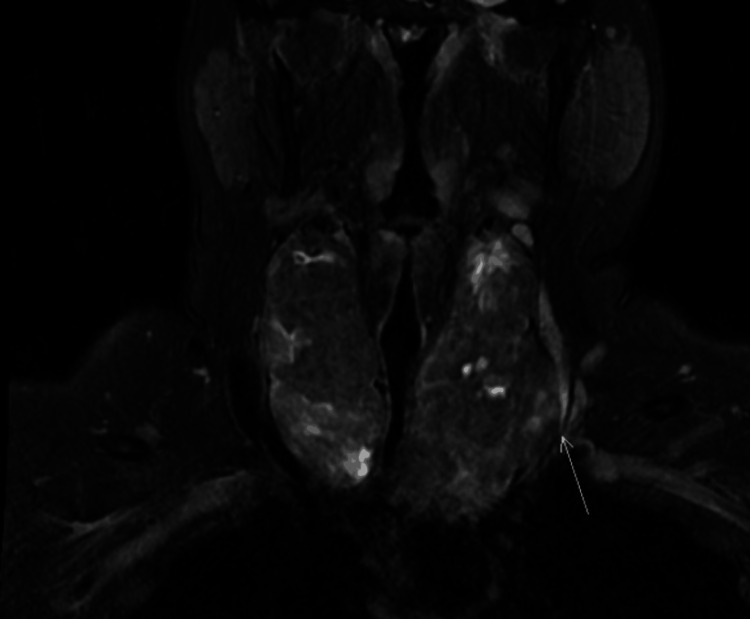
Large multinodular goiter with significant narrowing of the upper airways, with no evidence of malignancy Compression of the left internal jugular vein is shown by the arrow.

Initial imaging with CT thorax revealed a bilateral multinodular euthyroid goiter (118 mL volume) compressing the trachea to 3 mm at the narrowest point (Figure 1). MRI confirmed extrinsic tracheal stenosis without malignancy but with left internal jugular vein compression (Figure 2). Fiberoptic laryngoscopy demonstrated normal vocal cord mobility, and cervical ultrasound showed morphologically normal lymph nodes. Bronchoscopy was deferred due to stenosis-related bronchospasm risks. Laboratory results, including calcium and parathyroid hormone, were unremarkable.

Given the extrinsic tracheal compression and critical stenosis, a multidisciplinary team (endocrinology, anesthesiology, surgery) prioritized AFOI for airway management. Preemptive right femoral venovenous ECMO cannulation was performed as a contingency for airway collapse, and a hospital-employed Tamil interpreter was integrated into the team to ensure informed consent and real-time communication.

Intraoperatively, AFOI was performed under remifentanil target-controlled infusion (Ce of 3 ng/mL) and transcricoid lidocaine (2%, 3 mL). Initial desaturation (SpO₂ at 82%) during scope advancement resolved with jaw thrust, and a 6.5-mm endotracheal tube was secured on the second attempt. Hypertension during induction required urapidil, while hypotension post-induction necessitated norepinephrine infusion.

The patient underwent total thyroidectomy with parathyroid autotransplantation, preserving recurrent laryngeal nerves. Histopathology confirmed benign MNG. Postoperatively, persistent laryngopharyngeal edema delayed extubation (positive cuff leak test). ICU management included dexamethasone (8 mg q8h), high-flow oxygen, and heparin prophylaxis. Successful extubation occurred on postoperative day two, with SpO₂ of 96% on room air. Transient dysphagia required calcium supplementation, and levothyroxine was initiated for hypothyroidism.

At six-week follow-up, the patient was asymptomatic except for mild hoarseness. Endoscopy confirmed tracheal patency with minimal S-shaped curvature, and pulmonary function tests normalized. Calcium and parathyroid hormone levels stabilized with calcitriol adjustment.

## Discussion

Airway management in patients with MNG and severe tracheal stenosis presents significant anatomical and physiological challenges. In this case, AFOI proved indispensable for securing the airway while minimizing respiratory complications, aligning with Jung’s emphasis on AFOI’s role in high-risk anatomies [5]. The extrinsic tracheal compression (3 mm lumen) precluded videolaryngoscopy, which, while effective in intrinsic substernal goiters [6], risks displacing thyroid tissue into an already narrowed airway.

The decision to employ venovenous ECMO (V-V ECMO) as backup followed Behouche et al.’s protocol for compressive goiters [7], reflecting its growing role in difficult airway algorithms [8]. While ECMO was ultimately unnecessary, its preemptive use underscores its value in low-resource settings lacking emergent cardiopulmonary bypass. This contrasts with tertiary centers where ECMO is typically reserved for crises [8].

Interdisciplinary collaboration was pivotal to this case’s success. Though not explicitly detailed, our institutional adherence to checklists and standardized protocols, emphasized by Maldonado et al. [9], ensured seamless coordination between anesthesiologists, surgeons, and endocrinologists. Notably, the language barrier necessitated a hospital-employed Tamil interpreter, a resource absent in existing literature (Table 1) [2,6]. This highlights an ethical imperative: institutional policies must mandate interpreter availability to ensure equitable care [9].

**Table 1:** Comparison between our case and existing literature Source: Tasche et al. [2]

Aspect	Our Case	Literature
Intubation Method	AFOI + ECMO Backup	Videolaryngoscopy (97% sucess)
Stenosis Type	Extrinsic (3mm lumen)	Intrinsic (substernal goiter)
Barriers	Language + resources	None reported

AFOI’s operator dependence remains a limitation, requiring specialized training and equipment [5]. Future studies comparing AFOI with videolaryngoscopy in extrinsic stenosis could refine evidence-based guidelines, particularly for low-volume centers [8]. Furthermore, standardized protocols for interpreter integration in airway teams warrant broader advocacy to address systemic inequities in perioperative care [9].

## Conclusions

Airway management in MNG with severe tracheal stenosis demands a tailored approach that prioritizes anatomical realities and physiological risks. This case reaffirms AFOI as the gold standard for extrinsic tracheal compression, offering real-time airway assessment while preserving spontaneous respiration advantages unmatched by videolaryngoscopy in such scenarios. The preemptive use of venovenous ECMO as a contingency underscores its value in low-resource settings lacking emergent cardiopulmonary bypass, bridging the gap between tertiary and non-tertiary centers. Equally critical was the integration of interpreter-mediated communication, which ensured equitable consent and cooperation in a linguistically diverse patient, highlighting an often overlooked systemic gap in airway management protocols.

Multidisciplinary collaboration, spanning anesthesiology, surgery, and endocrinology, proved pivotal to navigating this high-risk airway, demonstrating that structured teamwork can mitigate operator-dependent limitations of AFOI. Moving forward, we advocate for the following: (1) protocol standardization for ECMO preparedness in extrinsic stenosis; (2) mandatory interpreter policies in institutional airway algorithms; and (3) comparative studies of AFOI versus videolaryngoscopy in resource-limited settings. This case exemplifies how adaptive strategies, rooted in anatomical precision and cultural competence, can optimize outcomes even in the most challenging clinical landscapes.
